# Association between clinic physician workforce and avoidable readmission: a retrospective database research

**DOI:** 10.1186/s12913-020-4966-4

**Published:** 2020-02-18

**Authors:** Yoshito Hirota, Susumu Kunisawa, Kiyohide Fushimi, Yuichi Imanaka

**Affiliations:** 1 Department of Healthcare Economics and Quality Management, Graduate School of Medicine, Kyoto University, Yoshida Konoecho, Sakyo-ku, Kyoto, 606-8501 Japan; 2 Department of Health Policy and Informatics, Graduate School of Medical and Dental Sciences, Tokyo Medical and Dental University, 1-5-45 Yushima, Bunkyo-ku, Tokyo, Japan

**Keywords:** Hospital readmission, Ambulatory care sensitive conditions, Clinic physician, Administrative database, Older adults

## Abstract

**Background:**

To reduce hospitalization costs, it is necessary to prevent avoidable hospitalization as well as avoidable readmission. This study aimed to examine the relationship between clinic physician workforce and unplanned readmission for ambulatory care sensitive conditions (ACSCs).

**Methods:**

The present study was a retrospective database research using nationwide administrative claims database of acute care hospitals in Japan. We identified patients aged ≥65 years who were admitted with ACSCs from home and discharged to home between April 2014 and December 2014 (*n* = 127,209). The primary outcome was unplanned readmission for ACSCs within 30 or 90 days of hospital discharge. A hierarchical logistic regression model was developed with patients at the first level and regions (secondary medical service areas) at the second level.

**Results:**

The 30-day and 90-day ACSC-related readmission rates were 3.7 and 4.6%, respectively. The high full-time equivalents (FTEs) of clinic physicians per 100,000 population were significantly associated with decreased odds ratios for 30-day and 90-day ACSC-related readmissions. This association did not change even when sensitivity analyses was conducted.

**Conclusions:**

Among patients who had history of admission for ACSCs, greater clinic physician workforce prevented the incidence of readmission because of ACSCs. Regional medical plans to prevent avoidable readmissions should incorporate policy interventions that focus on the clinic physician workforce.

## Background

The Japanese population is the most rapidly aging in the world [1]; approximately 28% of the population (or 35.6 million people) are aged ≥65 years [2]. The > 65 s account for 60% of the national medical care expenditure [3], and 86% of older adults have at least one chronic disease [4]. Hospitalization costs for chronic diseases and their complications are major contributors to increased medical expenses [5]. A previous study showed that the risk of avoidable hospitalization was higher among individuals who were aged ≥65 years compared with younger individuals [6]. The prevention of avoidable hospitalization of older adults is a viable strategy to reduce hospitalization costs.

The concept of ambulatory care sensitive conditions (ACSCs) is often used in the context of avoidable hospitalization [7]. ACSCs are defined as conditions for which appropriate outpatient care or early intervention (to prevent complications or more severe disease) can prevent the need for hospitalization [8]. ACSCs include diabetic complications, congestive heart failure, chronic obstructive pulmonary disease, bacterial pneumonia, and urinary tract infections.

Readmission is a common phenomenon and imposes a burden on the medical system [9]. In England and the United States, there is increasing impetus for efforts to prevent readmission for ACSCs [10]. Because appropriate outpatient care can prevent readmission for ACSCs, it is necessary to prevent both avoidable hospitalization and avoidable readmission.

Most previous studies that have examined the relationship between primary care physician per population and hospitalization for ACSCs have revealed lower hospitalization rates for ACSCs in areas with greater access to primary care [7]. However, to the best of our knowledge, no studies have examined the relationship between primary care physician workforce and readmission for ACSCs. Therefore, the primary objective of this study was to examine the relationship between the clinic physician workforce involved in primary care per population and unplanned readmission for ACSCs among older people.

## Methods

### Data source

The Diagnosis Procedure Combination (DPC) system is a case-mix classification system used in Japan for reimbursements to acute care hospitals under the public medical insurance scheme. We used data obtained from the DPC database which contains administrative claims data and discharge clinical summaries. The data were collected by the DPC research group from voluntarily participating hospitals, which account for approximately 50% of acute care hospitals in Japan [11]. The data were electronically collected through the uniform format stipulated by the Japanese Ministry of Health, Labour and Welfare for comparative analysis of many hospitals throughout Japan. The DPC database includes data pertaining to the following variables: hospital identifiers; patient demographics; zip code of the patient residence; major diagnoses, comorbidities present at admission and complications after admission recorded using the International Classification of Disease, 10th edition (ICD-10) codes; disease severity; length of stay; medications, surgical and interventional procedures with their specific dates of prescription and implementation; and discharge status. We also utilized data collected by the Ministry of Internal Affairs and Communications regarding the population, data collected by the Ministry of Land, Infrastructure, Transport and Tourism regarding residential area, and data collected by the Ministry of Health, Labour, and Welfare regarding the number of physicians and hospital beds.

### Subject inclusion and exclusion criteria

We identified patients who fulfilled the following criteria: 1) patients aged ≥65 years at the time of admission; 2) patients admitted unexpectedly with ACSCs between April 1 and December 31, 2014; 3) patients with no history of hospitalization within the past year; 4) patients admitted from home and discharged to home. Patients were followed up until March 31, 2015. For individuals who had ≥2 admissions during the study period, the first admission was considered as the index hospitalization. Patients with missing data pertaining to independent variables were excluded.

### Statistical analysis

The primary outcome variable was unplanned readmission for ACSCs within 30 days or 90 days of hospital discharge. ACSCs were those defined by Bardsley et al. [12]. Using 30-day or 90-day unplanned readmission for ACSCs as the dependent variables, a hierarchical logistic regression model was developed with patients at the first level and regions (secondary medical service areas) at the second level. We utilized a random intercept model with regions as random effects. A two-sided significance level of 0.05 was used, and all statistical analyses were performed using R software version 3.4.1 (R Foundation for Statistical Computing, Vienna, Austria).

### Patient-level variables

The patient-level variables were: age; sex; comorbidities; body mass index (BMI); Barthel index at discharge; surgery under general anesthesia, epidural anesthesia or spinal anesthesia; length of hospital stay; and schedule of implementation of home care program after discharge. Age was grouped into four categories: 65–74 years, 75–84 years, 85–94 years, and ≥ 95 years. Comorbidities were those defined by Elixhauser et al. [13]. Only those comorbid conditions that affected at least 1% of subjects were included. Consequently, the following 19 comorbid conditions were included: uncomplicated hypertension; uncomplicated and complicated diabetes; cardiac arrhythmias; congestive heart failure; chronic pulmonary disease; solid tumor without metastasis; fluid and electrolyte disorders; renal failure; peptic ulcer disease excluding bleeding; valvular disease; liver disease; deficiency anemia; peripheral vascular disorders; other neurological disorders; rheumatoid arthritis or collagen vascular diseases; blood loss anemia; hypothyroidism; and depression.

### Region-level variables

The region-level variables used were the full-time equivalents (FTEs) of clinic physicians per 100,000 population, the FTEs of hospital physicians per 100,000 population, the number of hospital beds per 100,000 population, and the population density of inhabitable areas (population/inhabitable area ratio) in the secondary medical service areas of residence of each subject. The secondary medical service areas are subprefectural regions comprising of several municipalities [14]. These government-stipulated regions are designed to provide comprehensive inpatient, outpatient, and long-term care in consideration of geographical conditions and access to necessities of daily life for residents. The region-level variables were represented by dummy variables indicating four quartiles in each of the secondary medical service areas, referring to a previous study [15]. We chose quartiles rather than a continuous variable due to the nonlinear relationship between the region-level variables and the outcomes. We also performed three sensitivity analyses. In the first sensitivity analysis, the region-level variables were represented by dummy variables indicating three tertiles and five quintiles in each of the secondary medical service areas instead of four quartiles. In the second sensitivity analysis, we used the number of clinic physicians per 100,000 population and the number of hospital physicians per 100,000 population in the secondary medical service areas instead of the respective FTEs. In the third sensitivity analysis, we restricted the target population to patients for whom the referral letter to clinic was issued during their hospital stay.

## Results

**Fig. 1:**
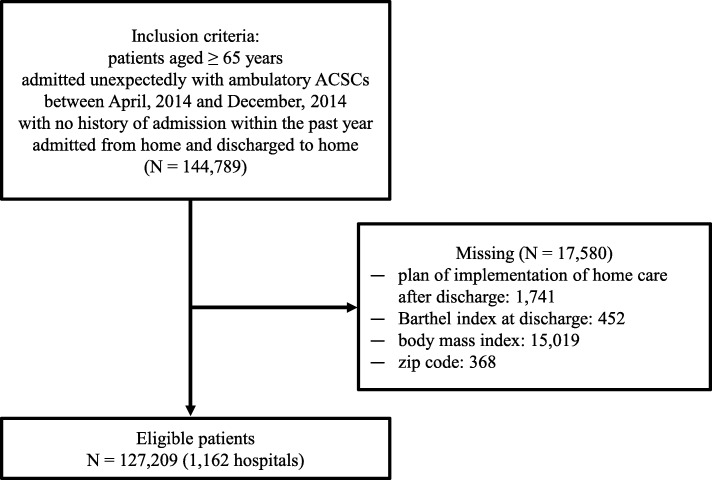
Schematic illustration of the patient selection criteria. Abbreviations: ACSCs, ambulatory care sensitive conditions

A total of 127,209 patients from 1162 hospitals and 344 secondary medical service areas were included in the analysis. Figure 1 shows a schematic illustration of the patient selection process. Additional file 1: Table S1 shows the thresholds defining the quartiles of region-level variables in 344 secondary medical service areas. Table 1 presents the descriptive statistics for the study variables. The 30-day and 90-day ACSC-related readmission rates were 3.7 and 4.6%, respectively. The mean age at index hospitalization was 78.3 (SD = 7.9) years; approximately 54% patients were male. Most patients were able to perform the activities of daily living (ADL) independently. Approximately 40% patients were affected by uncomplicated hypertension.

**Table 1:** Characteristics of the study population (*N* = 127,209)

Characteristic	*n* (%)	Median (IQR)
30-day ACSC-related readmission	4698 (3.7)	
90-day ACSC-related readmission	5842 (4.6)	
Age, year		78 (72–84)
Male	68,384 (53.8)	
BMI, kg/m^2^		22.2 (19.7–24.8)
Barthel index at discharge		100 (75–100)
Surgery	4071 (3.2)	
Length of stay, day		11 (7–19)
Plan of home care program after discharge	8337 (6.6)	
FTEs of clinic physicians per 100,000 population		99.0 (82.9–117.3)
FTEs of hospital physicians per 100,000 population		148.4 (117.4–204.0)
Number of hospital beds per 100,000 population		1223.0 (983.9–1568.4)
Population density of inhabitable areas, per km^2^		826.8 (390.0–2907.9)
Comorbidities		
Uncomplicated hypertension	49,546 (38.9)	
Complicated diabetes	21,141 (16.6)	
Uncomplicated diabetes	17,564 (13.8)	
Cardiac arrhythmias	16,731 (13.2)	
Congestive heart failure	12,609 (9.9)	
Chronic pulmonary disease	11,729 (9.2)	
Solid tumor without metastasis	8975 (7.1)	
Fluid and electrolyte disorders	8754 (6.9)	
Renal failure	7944 (6.2)	
Peptic ulcer disease excluding bleeding	5032 (4.0)	
Valvular disease	4828 (3.8)	
Liver disease	4670 (3.7)	
Deficiency anemia	3814 (3.0)	
Peripheral vascular disorders	3388 (2.7)	
Other neurological disorders	2779 (2.2)	
Rheumatoid arthritis	2591 (2.0)	
Blood loss anemia	1828 (1.4)	
Hypothyroidism	1609 (1.3)	
Depression	1578 (1.2)	

*Abbreviations*: *IQR* Interquartile range, *ACSC* Ambulatory care sensitive condition, *BMI* Body mass index, *FTEs* Full-time equivalents

Table 2 presents the results of hierarchical logistic regression analysis for 30-day and 90-day ACSC-related readmissions. The 3rd and 4th quartiles of FTEs of clinic physicians per 100,000 population were independently associated with decreased odds ratio for 30-day and 90-day ACSC-related readmissions. The following factors showed a significant association with increased risk of 30-day and 90-day ACSC-related readmissions: age (≥75 years), male sex, BMI < 18.5 kg/m^2^, low ADL function at discharge, length of stay, schedule of implementation of home care program after discharge, uncomplicated diabetes, complicated diabetes, cardiac arrhythmia, congestive heart failure, chronic pulmonary disease, solid tumor without metastasis, renal failure, valvular disease, liver disease, rheumatoid arthritis/collagen vascular diseases, and hypothyroidism (Additional file 1: Table S2).

**Table 2:** The risk of readmission for ACSCs associated with FTEs of clinic physicians per 100,000 population

	Readmissions for ACSCs within 30 days		Readmissions for ACSCs within 90 days	
	Adjusted odds ratio (95% CI)	*P*	Adjusted odds ratio (95% CI)	*P*
FTEs of clinic physicians per 100,000 population				
1st quartile	Reference		Reference	
2nd quartile	0.95 (0.85–1.06)	0.331	0.92 (0.83–1.02)	0.107
3rd quartile	0.86 (0.77–0.97)	0.013	0.84 (0.76–0.94)	0.002
4th quartile	0.87 (0.78–0.98)	0.024	0.86 (0.77–0.96)	0.007

Odds ratios were adjusted for age-group (65–74 years, 75–84 years, 85–94 years, and ≥ 95 years), gender, body mass index, Barthel Index at discharge, surgery, length of stay, plan of home care program at discharge, 19 comorbidities, FTEs of hospital physicians per 100,000 population, number of hospital beds per 100,000 population, and population density of inhabitable areas

*Abbreviations*: *ACSCs* Ambulatory care sensitive conditions, *FTEs* Full-time equivalents, *CI* Confidence interval

Tables 3 and 4 present the results of sensitivity analysis using region-level variables categorized into three tertiles and five quintiles in each of the secondary medical service areas instead of four quartiles, respectively. The high FTEs of clinic physicians per 100,000 population showed a significant association with a decreased risk of 30-day and 90-day ACSC-related readmissions.

**Table 3:** Results of sensitivity analysis in which the region-level variables were represented by dummy variables disaggregated by three tertiles in each of the secondary medical service areas

	Readmissions for ACSCs within 30 days		Readmissions for ACSCs within 90 days	
	Adjusted odds ratio (95% CI)	*P*	Adjusted odds ratio (95% CI)	*P*
FTEs of clinic physicians per 100,000 population				
1st tertile	Reference		Reference	
2nd tertile	0.91 (0.84–0.99)	0.021	0.90 (0.83–0.97)	0.004
3rd tertile	0.91 (0.82–1.01)	0.092	0.91 (0.83–1.01)	0.068

Odds ratios were adjusted for age groups (65–74 years, 75–84 years, 85–94 years, and ≥ 95 years), gender, body mass index, Barthel Index at discharge, surgery, length of stay, plan of home care program at discharge, 19 comorbidities, FTEs of hospital physicians per 100,000 population, number of hospital beds per 100,000 population, and population density of inhabitable areas.

*Abbreviations*: *ACSCs* Ambulatory care sensitive conditions, *FTEs* Full-time equivalents, *CI* Confidence interval

**Table 4:** Results of sensitivity analysis in which the region-level variables were represented by dummy variables disaggregated by five quintiles in each of the secondary medical service areas

	Readmissions for ACSCs within 30 days		Readmissions for ACSCs within 90 days	
	Adjusted odds ratio (95% CI)	*P*	Adjusted odds ratio (95% CI)	*P*
FTEs of clinic physicians per 100,000 population				
1st quintile	Reference		Reference	
2nd quintile	0.89 (0.79–1.01)	0.081	0.89 (0.79–1.00)	0.046
3rd quintile	0.91 (0.80–1.03)	0.141	0.88 (0.78–0.99)	0.028
4th quintile	0.80 (0.70–0.91)	0.001	0.81 (0.72–0.91)	0.001
5th quintile	0.85 (0.75–0.97)	0.017	0.85 (0.75–0.96)	0.007

Odds ratios were adjusted for age-group (65–74 years, 75–84 years, 85–94 years, and ≥ 95 years), gender, body mass index, Barthel Index at discharge, surgery, length of stay, plan of home care program at discharge, 19 comorbidities, FTEs of hospital physicians per 100,000 population, number of hospital beds per 100,000 population, and population density of inhabitable areas

*Abbreviations*: *ACSCs* Ambulatory care sensitive conditions, *FTEs* Full-time equivalents, *CI* Confidence interval

Table 5 presents the results of sensitivity analysis using the number of clinic physicians per 100,000 population and the number of hospital physicians per 100,000 population in the secondary medical service areas instead of their FTEs. The association between the number of clinic physicians per 100,000 population and the 30-day/90-day ACSC-related readmission became weaker but was statistically significant.

**Table 5:** Results of sensitivity analysis in which the number of clinic physicians per 100,000 population was used instead of their FTEs

	Readmissions for ACSCs within 30 days		Readmissions for ACSCs within 90 days	
	Adjusted odds ratio (95% CI)	*P*	Adjusted odds ratio (95% CI)	*P*
Number of clinic physicians per 100,000 population				
1st quintile	Reference		Reference	
2nd quintile	0.90 (0.80–1.02)	0.115	0.92 (0.82–1.03)	0.143
3rd quintile	0.94 (0.83–1.06)	0.321	0.95 (0.85–1.07)	0.378
4th quintile	0.87 (0.76–0.98)	0.023	0.88 (0.79–0.99)	0.036
5th quintile	0.86 (0.76–0.99)	0.029	0.88 (0.78–1.00)	0.046

Odds ratios were adjusted for age-group (65–74 years, 75–84 years, 85–94 years, and ≥ 95 years), gender, body mass index, Barthel Index at discharge, surgery, length of stay, plan of home care program at discharge, 19 comorbidities, FTEs of hospital physicians per 100,000 population, number of hospital beds per 100,000 population, and population density of inhabitable areas

*Abbreviations*: *ACSCs* Ambulatory care sensitive conditions, *FTEs* Full-time equivalents, *CI* Confidence interval

Table 6 presents the result of sensitivity analysis wherein the target population was restricted to patients for whom the referral letter to clinic was issued during their hospital stay. A total of 48,832 patients were included in this sensitivity analysis. The 4th quintile of FTEs of clinic physicians per 100,000 population was significantly associated with decreased risk of 30-day and 90-day ACSC-related readmissions.

**Table 6:** Results of sensitivity analysis in which the target population was restricted to patients for whom the referral letter to clinic was issued during their hospital stay (*N* = 48,832)

	Readmissions for ACSCs within 30 days		Readmissions for ACSCs within 90 days	
	Adjusted odds ratio (95% CI)	*P*	Adjusted odds ratio (95% CI)	*P*
FTEs of clinic physicians per 100,000 population				
1st quintile	Reference		Reference	
2nd quintile	0.99 (0.77–1.27)	0.943	1.03 (0.82–1.29)	0.807
3rd quintile	0.86 (0.67–1.11)	0.248	0.90 (0.72–1.12)	0.341
4th quintile	0.70 (0.54–0.91)	0.008	0.76 (0.60–0.96)	0.024
5th quintile	0.84 (0.65–1.08)	0.173	0.93 (0.74–1.17)	0.549

Odds ratios were adjusted for age-group (65–74 years, 75–84 years, 85–94 years, and ≥ 95 years), gender, body mass index, Barthel index at discharge, surgery, length of stay, plan of home care program at discharge, 19 comorbidities, FTEs of hospital physicians per 100,000 population, number of hospital beds per 100,000 population, and population density of inhabitable areas

*Abbreviations*: *ACSCs* Ambulatory care sensitive conditions, *FTEs* Full-time equivalents, *CI* Confidence interval

## Discussion

To the best of our knowledge, this is the first study to evaluate whether the FTEs of clinic physicians per 100,000 population affects the incidence of unplanned readmission for ACSCs. In this nationwide study, increase in clinic physician workforce was associated with a lower risk of readmission for ACSCs within 30 and 90 days. These findings did not change even in the sensitivity analyses. In previous studies, higher number of primary care physicians was associated with lower hospitalization rates for ACSCs [7]. In the present study, adequate availability of physicians involved in primary care was associated with lower risk of admission as well as readmission for ACSCs.

A systematic review examined the organizational aspects of primary care that contribute to the reduction in avoidable hospitalization; the results showed that adequate supply of primary care physicians and long-term relationship between primary care physicians and patients helped reduce hospitalization for chronic ACSCs [16]. Our study also suggests that long-term relationship between physicians and patients helps reduce readmission for ACSCs. In Japan, most hospitals have outpatient departments and physicians working at both hospitals and clinics provide primary care services. It is, therefore, necessary to take into consideration the primary care function of hospitals when examining the association between clinic physician workforce and readmission for ACSCs. According to the data collected by the Japanese Ministry of Health, Labour and Welfare (2014), the average number of clinic physicians per clinic was 1.3 [17]. Therefore, it is possible that regular patient visits to a specific clinic promote more robust physician-patient relationship as compared with regular visits to a specific hospital outpatient department. Consequently, patients who resided in an area with higher workforce of clinic physicians showed a lower risk of readmission for ACSCs in this study.

To take into consideration the primary care function of hospitals, we took two measures in our analyses. First, we included FTEs of hospital physicians per 100,000 population as explanatory variables. The FTEs of hospital physicians may reflect the workforce serving both inpatients and outpatients at hospitals. In our result, the negative association between FTEs of clinic physicians and readmission for ACSCs was identified even after adjusting for FTEs of hospital physicians. Second, we performed sensitivity analysis wherein we restricted the target population to patients whose referral letter was issued during their hospital stay. In most cases, the referral letter issued during hospitalization requests for ongoing outpatient care at the clinic where patient had regular visit before the hospitalization. In the sensitivity analysis, therefore, we restricted the target population to patients who regularly visited their clinic after discharge; the results revealed a negative association between FTEs of clinic physicians and readmission for ACSCs.

In the other sensitivity analysis, we used the number of clinic physicians per 100,000 population instead of their FTEs. The association between supply of clinic physicians and risk of readmission for ACSCs became weaker as compared with that observed with use of FTEs of clinic physicians per 100,000 population. A previous study identified a stronger association between FTEs of primary care physicians per 10,000 population and ACSC hospitalizations compared with number of primary care physicians per 10,000 population; in addition, FTEs of primary care physicians provided a more accurate reflection of the availability of primary care physician compared with their number [18]. These findings are consistent with our results.

Some limitations of our study should be acknowledged. First, because of the nature of the DPC database, our estimation of the risk of readmission was limited to patients who were re-hospitalized at the same hospital as the index hospitalization. Therefore, the risk of readmission may have been underestimated. However, according to the study conducted at a Japanese hospital, the readmission rate of older patients with heart failure within 30 days and 1 year was 6–8% and 18–23%, respectively [19]. In another study based on data from the Japanese cardiac registry of heart failure in cardiology (JCARE-CARD), the readmission rate of patients with chronic heart failure for acute exacerbation within 1 year was approximately 25% [20]. In this DPC database, readmission rate of patients with heart failure within 30 days and 1 year was 6.6 and 22.8%, respectively (data not shown). Consequently, our estimation of the risk of readmission seems reasonable. Second, we utilized clinic physician workforce as covariates; however, we could not determine the number of clinic physicians disaggregated by specialty from the data collected by the Ministry of Health, Labour and Welfare. Although we could not use the number of physicians specializing in general internal medicine or family medicine as covariates, ACSCs include diseases that require treatment in other clinical departments, such as dermatology, otorhinolaryngology, obstetrics and gynecology, or dentistry [12]. Furthermore, there is no general practitioner system in Japan and most physicians become specialists [21]. Therefore, primary care is often provided by different specialists [22]. Consequently, the utilization of clinic physician workforce as covariates may be acceptable. Third, information about patients’ families and caregivers was not available from the database. The caregivers’ ability to care is liable to influence the risk of readmission and may have confounded our results.

## Conclusions

We found that in patients who had history of admission for ACSCs, larger clinic physician workforce prevented the occurrence of readmission for ACSCs. In previous studies, larger primary care physician workforce was shown to be associated with lower risk of hospitalization for ACSCs. In our study, adequate availability of physicians involved in primary care was associated with a lower risk of not only admission but also readmission for ACSCs. Further studies should investigate the quality and the continuity of primary care rather than just workforce.

## Supplementary information

**Additional file 1: Table S1.** Characteristics of Secondary Medical Service Areas (*N* = 344). Secondary medical areas are subprefectural regions comprising of several municipalities. Abbreviations: FTEs, full-time equivalents. **Table S2.** Results of hierarchical logistic regression showing correlates of 30-day and 90-day ACSC-related readmissions. Abbreviations: ACSCs, ambulatory care sensitive conditions; FTEs, full-time equivalents; CI, confidence interval; BMI, body mass index.

## Data Availability

The data that support the findings of this study are available from the DPC research group but restrictions apply to the availability of these data, which were used under license for the current study, and so are not publicly available. Data are, however, available from the authors upon reasonable request and with permission of the DPC research group.

## Abbreviations

ACSCs: Ambulatory care sensitive conditions
ADL: Activities of daily living
BMI: Body mass index
CI: Confidence interval
DPC database: Diagnosis procedure combination database
FTEs: Full-time equivalents
ICD-10: International classification of disease, 10th edition
IQR: Interquartile range
JCARE-CARD: Japanese cardiac registry of heart failure in cardiology
SD: Standard deviation
