# Supplementary material for: Association between clinic physician workforce and avoidable readmission: a retrospective database research
Source: BMC Health Serv Res. 2020 Feb 18;20:125. doi: 10.1186/s12913-020-4966-4 (PMC7029440; doi:10.1186/s12913-020-4966-4)
Supplement: Supplementary file 1 — Additional file 1: Table S1. Characteristics of Secondary Medical Service Areas (N = 344). Secondary medical areas are subprefectural regions comprising of several municipalities. Abbreviations: FTEs, full-time equivalents. Table S2. Results of hierarchical logistic regression showing correlates of 30-day and 90-day ACSC-related readmissions. Abbreviations: ACSCs, ambulatory care sensitive conditions; FTEs, full-time equivalents; CI, confidence interval; BMI, body mass index. [file 12913_2020_4966_MOESM1_ESM.docx]

| Additional Table 1. Characteristics of secondary medical service areas (N = 344) | |
| --- | --- |
| Characteristic | Value |
| FTEs of clinic physicians per 100,000 population |  |
| 1st quartile | 72.3 |
| 2nd quartile | 87.6 |
| 3rd quartile | 102.6 |
| 4th quartile | 481.1 |
| FTEs of hospital physicians per 100,000 population |  |
| 1st quartile | 103.7 |
| 2nd quartile | 128.1 |
| 3rd quartile | 155.0 |
| 4th quartile | 807.1 |
| Number of hospital beds per 100,000 population |  |
| 1st quartile | 1025.0 |
| 2nd quartile | 1283.8 |
| 3rd quartile | 1632.3 |
| 4th quartile | 3539.5 |
| Population density of inhabitable areas, per km² |  |
| 1st quartile | 196.4 |
| 2nd quartile | 365.9 |
| 3rd quartile | 713.9 |
| 4th quartile | 8777.8 |

Secondary medical areas are subprefectural regions comprising of several municipalities.

Abbreviations: FTEs, full-time equivalents

| **Additional Table 2. Results of hierarchical logistic regression showing correlates of 30-day and 90-day ACSC-related readmissions** | | | | |  |
| --- | --- | --- | --- | --- | --- |
|  | Readmissions for ACSCs within 30 days | | Readmissions for ACSCs within 90 days | |  |
| Variables | Adjusted odds ratio (95% CI) | P | Adjusted odds ratio (95% CI) | P |  |
| Age, years |  |  |  |  |  |
| 65–74 | Reference |  | Reference |  |  |
| 75–84 | 1.26 (1.17–1.36) | <0.001 | 1.25 (1.17–1.33) | <0.001 |  |
| 85–94 | 1.50 (1.38–1.63) | <0.001 | 1.50 (1.39–1.62) | <0.001 | |
| ≥95 | 1.87 (1.55–2.26) | <0.001 | 1.85 (1.56–2.20) | <0.001 |  |
| Male | 1.10 (1.03–1.17) | 0.002 | 1.09 (1.03–1.15) | 0.004 |  |
| BMI, kg/m² |  |  |  |  |  |
| ≥18.5 and <25 | Reference |  | Reference |  |  |
| <18.5 | 1.18 (1.09–1.27) | <0.001 | 1.18 (1.10–1.27) | <0.001 |  |
| ≥25 | 0.89 (0.83–0.96) | 0.002 | 0.91 (0.85–0.97) | 0.005 |  |
| Barthel index at discharge |  |  |  |  |  |
| ≥85 | Reference |  | Reference |  |  |
| ≥75 and <85 | 1.24 (1.08–1.41) | 0.002 | 1.26 (1.12–1.42) | <0.001 |  |
| ≥60 and <75 | 1.20 (1.07–1.35) | 0.002 | 1.27 (1.15–1.41) | <0.001 |  |
| ≥50 and <60 | 1.32 (1.17–1.49) | <0.001 | 1.42 (1.27–1.57) | <0.001 |  |
| ≥40 and <50 | 1.47 (1.26–1.71) | <0.001 | 1.45 (1.27–1.67) | <0.001 |  |
| <40 | 1.54 (1.40–1.69) | <0.001 | 1.50 (1.37–1.64) | <0.001 |  |
| Surgery | 0.58 (0.48–0.70) | <0.001 | 0.52 (0.44–0.63) | <0.001 |  |
| Length of stay | 1.01 (1.01–1.01) | <0.001 | 1.01 (1.01–1.01) | <0.001 |  |
| Plan of implementation of home care program after discharge | 1.13 (1.02–1.06) | 0.022 | 1.13 (1.03–1.25) | 0.011 |  |
| Comorbidities |  |  |  |  |  |
| Uncomplicated  　　　hypertension | 1.06 (0.99–1.12) | 0.077 | 1.07 (1.01–1.13) | 0.020 |  |
| Complicated diabetes | 1.84 (1.71–1.98) | <0.001 | 1.91 (1.79–2.04) | <0.001 |  |
| Uncomplicated diabetes | 1.09 (1.00–1.20) | 0.058 | 1.11 (1.02–1.21) | 0.012 |  |
| Cardiac arrhythmias | 1.32 (1.21–1.43) | <0.001 | 1.33 (1.23–1.43) | <0.001 |  |
| Congestive heart failure | 1.16 (1.06–1.27) | 0.002 | 1.12 (1.03–1.22) | 0.006 |  |
| Chronic pulmonary  disease | 1.36 (1.24–1.50) | <0.001 | 1.41 (1.30–1.54) | <0.001 |  |
| Solid tumor without  　　　metastasis | 1.28 (1.15–1.43) | <0.001 | 1.20 (1.08–1.32) | <0.001 |  |
| Fluid and electrolyte  　　　disorders | 0.90 (0.80–1.02) | 0.103 | 0.91 (0.82–1.02) | 0.094 |  |
| Renal failure | 1.53 (1.38–1.69) | <0.001 | 1.55 (1.42–1.69) | <0.001 |  |
| Peptic ulcer disease  　　　excluding bleeding | 1.03 (0.88–1.20) | 0.720 | 1.01 (0.88–1.16) | 0.903 |  |
| Valvular disease | 1.23 (1.07–1.41) | 0.004 | 1.30 (1.15–1.47) | <0.001 |  |
| Liver disease | 1.34 (1.15–1.55) | <0.001 | 1.29 (1.12–1.47) | <0.001 |  |
| Deficiency anemia | 0.85 (0.70–1.02) | 0.085 | 0.92 (0.78–1.08) | 0.319 |  |
| Peripheral vascular  　　　disorders | 1.11 (0.93–1.32) | 0.236 | 1.11 (0.95–1.30) | 0.181 |  |
| Other neurological  　　　disorders | 1.13 (0.93–1.36) | 0.219 | 1.08 (0.90–1.28) | 0.402 |  |
| Rheumatoid arthritis | 1.02 (0.82–1.26) | 0.886 | 1.13 (0.94–1.36) | 0.198 |  |
| Blood loss anemia | 0.74 (0.55–1.01) | 0.062 | 0.64 (0.48–0.87) | 0.004 |  |
| Hypothyroidism | 1.32 (1.05–1.66) | 0.018 | 1.44 (1.17–1.75) | <0.001 |  |
| Depression | 0.87 (0.65–1.16) | 0.329 | 0.93 (0.72–1.20) | 0.564 |  |
| FTEs of clinic physicians per 100,000 population |  |  |  |  |  |
| 1st quartile | Reference |  | Reference |  |  |
| 2nd quartile | 0.95 (0.85–1.06) | 0.331 | 0.92 (0.83–1.02) | 0.107 |  |
| 3rd quartile | 0.86 (0.77–0.97) | 0.013 | 0.84 (0.76–0.94) | 0.002 |  |
| 4th quartile | 0.87 (0.78–0.98) | 0.024 | 0.86 (0.77–0.96) | 0.007 |  |
| FTEs of hospital physicians per 100,000 population |  |  |  |  |  |
| 1st quartile | Reference |  | Reference |  |  |
| 2nd quartile | 1.10 (0.99–1.24) | 0.089 | 1.12 (1.01–1.24) | 0.036 |  |
| 3rd quartile | 1.05 (0.93–1.18) | 0.448 | 1.10 (0.98–1.23) | 0.097 |  |
| 4th quartile | 1.11 (0.98–1.25) | 0.104 | 1.12 (1.00–1.26) | 0.049 |  |
| Number of hospital beds per 100,000 population |  |  |  |  |  |
| 1st quartile | Reference |  | Reference |  |  |
| 2nd quartile | 0.99 (0.90–1.09) | 0.874 | 0.98 (0.90–1.07) | 0.696 |  |
| 3rd quartile | 1.01 (0.91–1.13) | 0.842 | 0.98 (0.89–1.09) | 0.727 |  |
| 4th quartile | 1.02 (0.90–1.15) | 0.763 | 1.00 (0.90–1.12) | 0.950 |  |
| Population density of inhabitable areas, /km² |  |  |  |  |  |
| 1st quartile | Reference |  | Reference |  |  |
| 2nd quartile | 1.01 (0.89–1.16) | 0.839 | 0.98 (0.87–1.11) | 0.770 |  |
| 3rd quartile | 1.00 (0.88–1.13) | 0.978 | 0.98 (0.87–1.10) | 0.748 |  |
| 4th quartile | 1.02 (0.90–1.16) | 0.768 | 1.01 (0.89–1.13) | 0.910 |  |
| C-statics (95% CI) | 0.638 (0.630–0.646) | | 0.643 (0.636–0.650) | |  |

Abbreviations: ACSCs, ambulatory care sensitive conditions; FTEs, full-time equivalents; CI, confidence interval; BMI, body mass index
